# Epidermoid cyst of an intrapancreatic accessory spleen: a case report and literature review

**DOI:** 10.1186/1477-7819-12-92

**Published:** 2014-04-10

**Authors:** Nick Zavras, Nick Machairas, Pericles Foukas, Andreas Lazaris, Paul Patapis, Anastasios Machairas

**Affiliations:** 1 Third Department of Surgery, ATTIKO University Hospital, Athens, Greece; 2 Second Department of Surgery, Laiko University Hospital, Athens, Greece; 3 Second Department of Pathology, ATTIKO University Hospital, Athens, Greece

## Abstract

**Background:**

An epidermoid cyst in an intrapancreatic accessory spleen is a rare lesion. Despite advances in radiologic techniques, in most cases it has been diagnosed preoperatively as a possible pancreatic neoplasm.

**Case presentation:**

Herein, we present a 63-year-old Caucasian woman, diagnosed preoperatively with enhanced-contrast abdominal computed tomography, as having a potential cystic tumor in the tail of the pancreas. The patient underwent a distal pancreatectomy and splenectomy, and the histological examination revealed the presence of an epidermoid cyst of an accessory intrapancreatic spleen.

**Conclusions:**

Familiarity with the imaging features, the clinical presentation and the location of the cyst are important to consider if this rare entity is to be included in the differential diagnosis of cystic neoplasms of the pancreas.

## Background

The presence of an accessory spleen (AS) at autopsy is estimated to be about 10%, almost 20% of which are found in or attached to the tail of the pancreas [1,2]. Epidermoid cysts (ECs) account for 10% of benign non-parasitic cysts of the spleen [3]. However, the presence of an EC in an AS is very rare, with 33 cases of ECs found in an intrapancreatic (IP) AS [4-36], and only one in an AS located in the greater omentum [3].

Herein, we report on a case of an epidermoid cyst in intrapancreatic accessory spleen (ECIPAS), and make a comprehensive review of the literature.

## Case presentation

**Figure 1:**
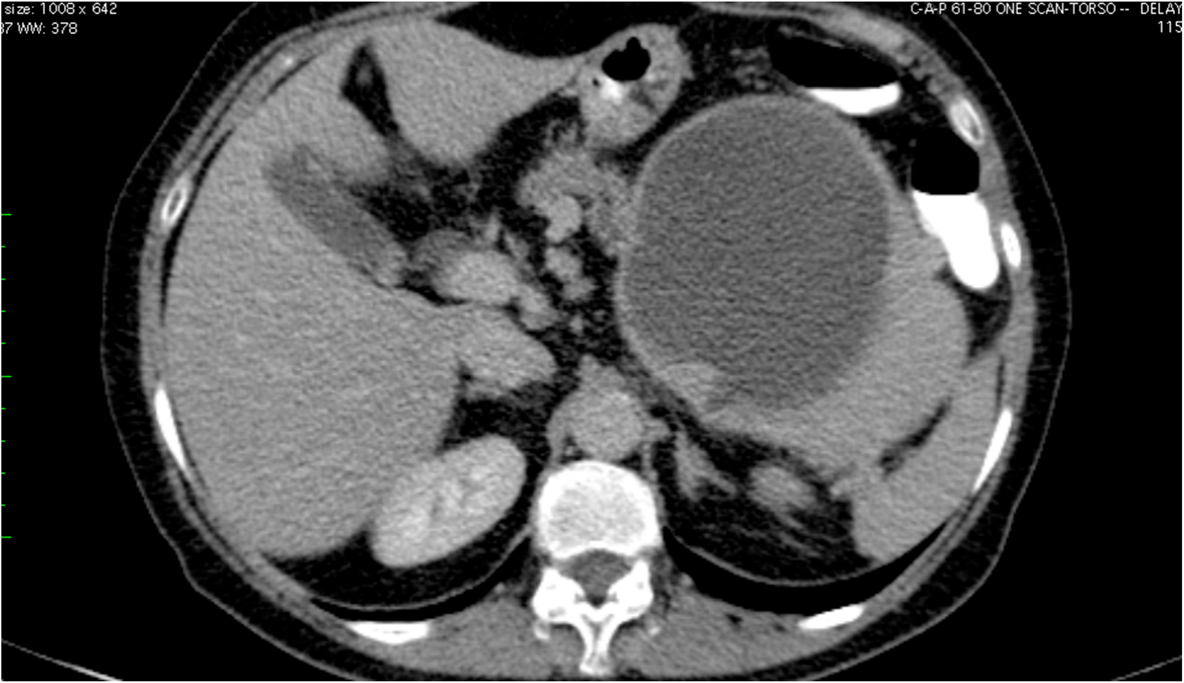
Enhanced computed tomography (CT) shows the presence of a cystic lesion in the tail of the pancreas.

A 63-year-old Caucasian woman was admitted to our hospital with a one-week history of nausea and vomiting after meals. Her medical history included surgery for a peptic ulcer at the age of 48 years. Physical examination was essentially unremarkable. Laboratory data showed normal values. Enhanced-contrast abdominal computed tomography (CT) revealed a mass lesion with solid and cystic components detected in the tail of the pancreas (Figure 1). As concerns serum tumor markers, carbohydrate antigen (CA) 19-9 levels had increased to 222 U/ml (reference range 0 to 27 U/ml). Because a malignant tumor of the pancreas was suspected, the patient underwent a distal pancreatectomy and splenectomy.

**Figure 2:**
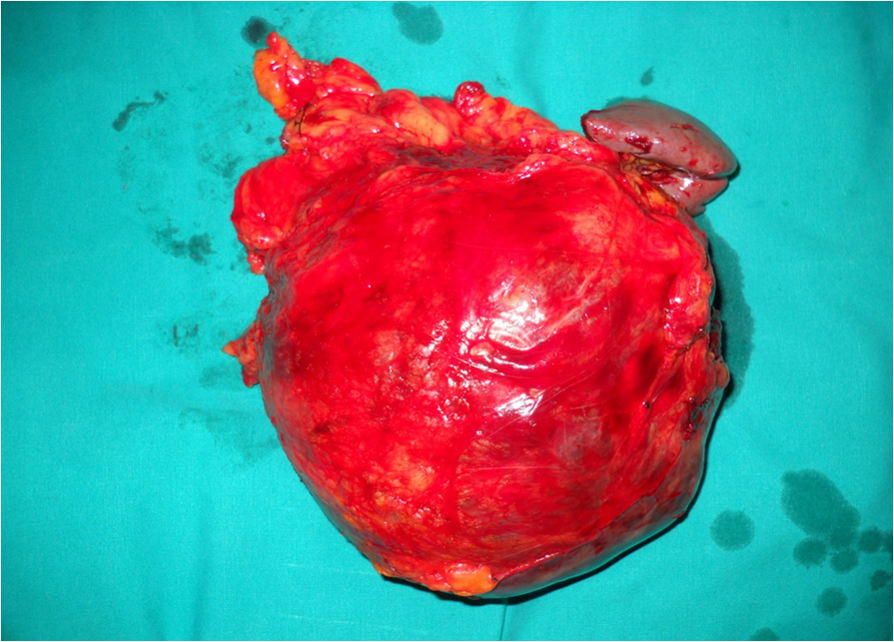
Gross appearance of the epidermoid cyst in intrapancreatic accessory spleen (ECIPAS), with 12.6 cm at its greatest diameter.

**Figure 3:**
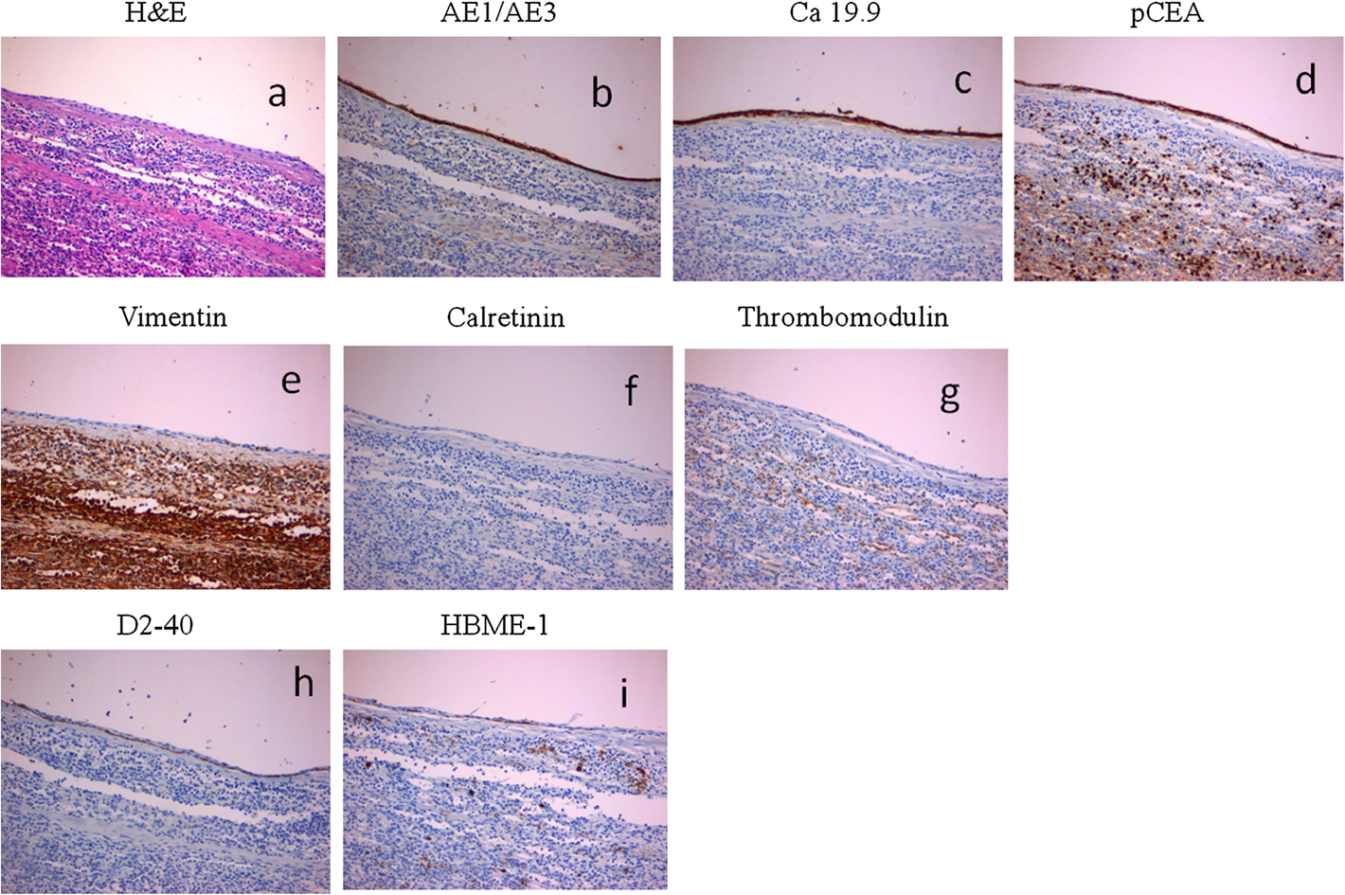
**Histopathological findings.** Microscopically, the lining epithelium in this area **(a)** consists of two to three layers thick squamous flattened epithelium above a red pulp splenic parenchyma (H&E, x20). Immunohistochemical characteristics of the lining epithelium: note positive staining for **(b)** keratins AE1/AE3, **(c)** CA 19-9, **(d)** pCEA, absence of reactivity for **(e)** vimentin, **(f)** calretinin, and **(g)** thrombomodulin, and focal positivity for **(h)** D2-40 and **(i)** HBME-1 (H&E, x20).

The cyst measured 12.6 cm at its greatest diameter (Figure 2), and contained a brownish serous composition fluid. No hair or skin appendages were found. Biochemical analysis of the cystic fluid revealed a markedly high level of CA 19-9 (5,000 U/mL) and a moderate elevation of CEA (180.4 ng/ml). Microscopically, the cyst was lined with multilayered (two to five layers thick) flattened epithelium, reminiscent of squamous epithelium above a red pulp splenic parenchyma (Figure 3a). Immunohistochemistry showed that the epithelial cells were positive for keratins AE1/AE3 (Figure 3b), CA 19-9 (Figure 3c) and pCEA (Figure 3d) and negative for vimentin, calretinin and thrombomodulin. Basal epithelial cells where focally reactive with antibodies against D2-40 (Figure 3e) and HBME-1 (Figure 3f). The pathological diagnosis indicated a true epithelial cyst of IPAS.

Postoperatively, CA 19-9 serum decreased to normal levels after one and a half months. One year later, the patient remains in good health.

## Discussion

In 1980, Davidson *et al*. [4] reported the first case of ECIPAS; and since then 33 articles and 36 patients have been reported in the English language literature (Table 1), suggesting the rarity of the disease. However, the exact incidence of ECIPAS is difficult to determine as over 50% of the cases were incidentally detected [8,9,12,14,15,18,19,22-24], [26,27,30,32,34]. The mean age of the patients was 46.1 years (range 12 to 70 years), with a female preponderance (58.3%) [6,7,10-13,16,17,19-21,24-26,28], [31,33,34,36]. It is noteworthy that the majority of patients were of Asian origin (28/36 patients, 77.7%), suggesting possibly the presence of a racial factor [26,27].

**Table 1:** List of all published cases of epidermoid cyst in intrapancreatic accessory spleen (ECIPAS) in the English language literature

**Case**	**Author**	**Sex**	**Age (years)**	**Presentation**	**Location**	**Size (cm)**	**Serum markers**		**Preoperative diagnosis**	**Surgery**
							**CEA**	**CA 19-9**		
1	Davidson *et al*., 1980 [4]	Male	40	Nausea, WL, chest pain	Tail	5.5	NI	NI	Pseudocyst, cystadenoma, cystadenocarcinoma	DP/SPL
2	Hanada *et al*., 1981 [5]	Male	51	RLQ	Tail	6	NI	NI	Pseudocyst	DP/SPL
3	Morodoshi *et al*., 1991 [6]	Female	32	Left abdominal pain	Tail	6	Normal	Normal	Pancreatic cyst	Cyst removal
4	Nakae *et al*., 1991 [7]	Female	37	Epigastric pain	Tail	6.5	NI	NI	Pancreatic cyst	Spleen preserving DP
5	Tang *et al*., 1994 [8]	Male	38	Asymptomatic	Tail	1.4	NI	NI	-	DP/SPL
6	Furukawa *et al*., 1998 [9]	Male	45	Asymptomatic	Tail	2.0	NI	NI	Primary cystic neoplasm	DP
7	Higaki *et al*., 1998 [10]	Female	46	Left back pain	Tail	-	NI	+	Malignant tumor	DP/SPL
8	Tateyama *et al*., 1998 [11]	Female	67	Abdominal fullness, intermittent upper abdominal pain	Tail	3.0	+	+	-	DP/SPL
9	Sasou *et al*., 1999 [12]	Female	49	Asymptomatic	Tail	4.3	NI	NI	Cystic tumor of the pancreas	DP/SPL
10	Choi *et al*., 2000 [13]	Female	54	Epigastric pain, nausea, vomiting, WL	Tail	15	NI	NI	Benign cyst of the pancreas or AS	DP/SPL
11	Tsutsumi *et al*., 2000 [14]	Male	51	Asymptomatic	Tail	51	Normal	Normal	Benign cyst of the pancreas	DP/SPL
12	Horibe *et al*., 2001 [15]	Male	48	Asymptomatic	Tail	2.0	-	+	MCN producing pancreatic tumor	DP/SPL
13	Sonomura *et al*., 2002 [16]	Female	45	Epigastric pain	Tail	3.5	-	-	Cystadenocarcinoma or solid tumor of the pancreas	DP/SPL
14	Fink *et al*., 2002 [17]	Female	12	Fever	Tail	2.0	-	-	Infected abdominal cyst	Cyst removal
15	Yokomizo *et al.*, 2002 [18]	Male	38	Asymptomatic	Tail	3.0	-	++	Mucinous cystadenoma, adenocarcinoma, ECIPAS	DP/SPL
16	Kanazawa *et al*., 2004 [19]	Female	58	Asymptomatic	Tail	2.5	-	+	MCN	Spleen preserving DP
17	Watanabe *et al*., 2004 [20]	Female	55	Postprandial epigastralgia	Tail	3	Normal	++	Mucinous cystadenoma, cystadenocarcinoma	DP/SPL
18	Won *et al*., 2005 [21]	Male	32	Asymptomatic	Tail	7.5	NI	+	Pancreatic pseudocyst	Spleen preserving DP
	Won *et al*., 2005 [21]	Female	49	LUQ abdominal pain	Tail	2.0	Normal	Normal	Serous or MCN cystadenoma	Laparoscopic DP
19	Ru *et al*., 2007 [22]	Male	41	Asymptomatic	Tail	2.5	NI	-	Cystic lesion of the pancreas	DP/SPL
20	Itano *et al*., 2008 [23]	Male	40	Asymptomatic	Tail	4.0	Normal	Normal	ECIPAS	DP/SPL
21	Servais *et al*., 2008 [24]	Female	52	Asymptomatic	Tail	10.0	+	+	Malignant pancreatic neoplasm	DP/SPL
22	Gleeson *et al*., 2008 [25]	Female	32	RUQ pain	Tail	1.5	-	-	Cystic pancreatic neoplasm	DP/SPL
23	Zhang *et al*., 2009 [26]	Female	26	Asymptomatic	Tail	2.5	Normal	Normal	Primary MCN	Spleen preserving DP
24	Reiss *et al*., 2009 [27]	Male	49	Asymptomatic	Tail	3.6	NI	NI	MCN	DP/SPL
25	Kadota *et al*., 2010 [28]	Female	57	Asymptomatic	Tail	2.6	Normal	Normal	Pancreatic cystic tumor	DP
	Kadota *et al*., 2010 [28]	Female	70	Asymptomatic	Tail	6.0	Normal	Normal	MCN	DP/SPL
	Kadota *et al*., 2010 [28]	Male	37	Asymptomatic	Tail	2.6	Normal	Normal	Serous cystic tumor or lymphoepithelial cyst	DP
26	Itano *et al*., 2010 [29]	Male	67	Epigastric pain, WL	Tail	2.2	NI	+	ECIPAS	Laparoscopic DP /SPL
27	Horn *et al*., 2011 [30]	Male	62	Abdominal pain left-sided	Tail	4.8	NI	NI	Retroperitoneal left-sided cystic mass	Cyst removal
28	Iwasaki *et al*., 2011 [32]	Female	36	Asymptomatic	Tail	3.4	NI	+	MCN	Laparoscopic DP/SPL
29	Yamanishi *et al*., 2011 [31]	Female	55	Asymptomatic	Tail	3.3	N	+	MCN	DP
30	Urakami *et al*., 2011 [33]	Female	50	Asymptomatic	Tail	3.0	NI	NI	ECIPAS or other cystic tumor in IPAS	Laparoscopic spleen preserving DP
31	Khashab *et al*., 2011 [34]	Female	49	Nonspecific abdominal pain	Tail	2.3	NI	NI	PNET	Laparoscopic spleen preserving DP
32	Harris *et al*., 2012 [35]	Female	39	Asymptomatic	Tail	2.0	+	NI	Malignant cystic tumor	Laparoscopic DP/SPL
33	Hong *et al*., 2013 [36]	Female	54	Abdominal discomfort	Tail	2.3	NI	NI	NI	Spleen preserving DP
34	Our patient	Female	63	Nausea, vomiting	Tail	12.6	Normal	+		DP/SPL

WL: weight loss, NI: no information, DP: distal pancreatectomy, SPL: splenctomy, RLQ: Right lower quadrant, MCN: Mucinous neoplasm, LUQ: Left upper quadrant, PNET: Pancreatic neuroendocrine tumor.

The precise histogenesis of an ECIPAS is not well understood. In summarizing the results of the literature, three main theories have been proposed. The first is based on similar studies of the histogenesis of ECs in the normal spleen suggesting an invagination of capsular mesothelium with subsequent cystic formation and metaplastic changes [37,38]. The second, based on the presence of keratokine profile of a splenic cyst advocated that ECs are of teratomatous derivation or from inclusion of fetal squamous epithelium [39]. The third, based on immunohistochemical findings, suggests that an ECIPAS may derive either from an aberrant embryonic inclusion of the pancreatic duct epithelium [6], or from a protrusion of a pancreatic duct into an IPAS [11]. The later is questionable as macroscopically, Yokomizo *et al*. [18] and Iwasaki *et al*. [31] by using retrograde pancreatography, and Urakami *et al*. [32] by using magnetic resonance cholangiopancreatography, found no relationship between the pancreatic duct and the ECIPAS.

The histological findings of an ECIPAS in most cases demonstrate a unilocular or multilocular cyst lined by stratified squamous epithelium, keratinizing or not, and surrounded by normal splenic tissue [4-17,19-23,26-29,32-34]. No skin appendages have been identified [4-34]. Immunohistochemical examinations of lining epithelium demonstrate positivity for CA 19-9 [6,10-12,15,18,20,22,24,27], [30] and CEA [10-12,15,22,24,29]. Our immunohistochemical findings were found to accord with those of the above mentioned studies, showing positivity for anti-CA 19-9 and anti-pCEA antibodies. According to Higaki *et al*. [10], the high levels of CA 19-9 and/or CEA in the serum and in the cystic fluid [11] are produced by the squamous epithelium lining and released into the circulation due to trauma or increased intracystic pressure. The fall in levels noted after surgery further supports this suggestion [10].

The clinical presentation is not characteristic. Symptoms include epigastric pain, abdominal pain/discomfort, nausea, vomiting, and weight loss. However, in the reviewed cases, twenty patients (58.3%) (Table 1) were asymptomatic and were identified during radiological examinations for other reasons.

At present, U/S, CT-scan, and MRI are the main imaging tools to detect the lesion. The diameter of the cyst in the reported cases varied from 1.4 to 15 cm (mean 3.89 cm, 2.66 SD). On MRI, the cystic component was hypointense on T1-weighted images and hyperintense on T2-weighted images [13,18-20,23,29,33]. However, in most cases a diagnosis of a mucinous cystadenoma [4,15,18-21,26,27,30,31], cystadenocarcinoma [4,16,18,20], pseudocyst [4,5,17], or a potential malignant tumor [25,34] was suspected. Interestingly, in two cases [23,29], an ECIPAS was diagnosed in one [23] and strongly suspected in the other [29] based on CT and MRI findings. The radiological signs were related to the homogeneous attenuation of the solid component of the cyst and the adjacent spleen on enhanced CT studies and T1-weighted magnetic resonance images, and on the smooth cystic nature of the inner wall. Itano *et al*. [23] stated that a relatively adequate splenic mass of AS surrounding the EC is essential for a correct preoperative diagnosis. Additional diagnostic modalities such as endoscopic ultrasonography (EUS), EUS-guided fine needle aspiration of the cystic component, fluorine-18 fluorodeoxyglucose positron emission tomography (FDG-PET) [31], and EUS- elastography [33] may be used as complementary tools in the diagnosis of an ECIPAS. Recently, a promising diagnostic method was suggested by Motosugi *et al*. [40], who reported on five subjects as having ECIPAS by using superparamagnetic iron oxide enhanced MRI. Four of them were followed up without surgical intervention.

Although the lesion is considered to be benign and surgery was avoidable [41] Elit *et al*. [42] reported a squamous cell carcinoma deriving from an EC located in the normal spleen. Taking into account that the lesions of the normal spleen could affect an AS [5], a possible malignant transformation cannot be excluded if the cyst remains unresected. However, no malignancy of an ECIPAS has yet been reported.

Until now, the treatment of ECIPAS consists of surgical removal, either open [4-28,30-32,36], or laparoscopic [29,33-35], with or without splenic preservation. No death has been reported during operation or in the short-term postoperative period.

## Conclusions

An ECIPAS is a very rare entity. So far, there are not accurate criteria for the preoperative diagnosis of an ECIPAS, and a definite diagnosis derives from pathological examination after surgical removal. Advances in imaging techniques and familiarity with the radiological findings and clinical characteristics of ECIPAS may help determine the correct management of this lesion.

## Consent

Written informed consent was obtained from the patient for publication of this case and for the accompanying images.

## Abbreviations

AS: accessory spleen; CA: carbohydrate antigen; CEA: carcinoembryonic antigen; CT: computed tomography; EC: epidermoid cyst; ECIPAS: epidermoid cyst in intrapancreatic accessory spleen; EUS: endoscopic ultrasonography; FDG-PET: fluorine-18 fluorodeoxyglucose positron emission tomography; H&E: hematoxylin and eosin; MRI: magnetic resonance imaging.

## Competing interests

All authors have made substantive contributions to the study, and are in agreement with the conclusions of the study. Furthermore, there are no financial competing interests.

## Authors’ contributions

NZ and NM wrote the paper. PF carried out the histological and immunohistochemical studies of the surgical specimens. AM, PP and AL were involved in the preoperative, intraoperative and postoperative management of the patient. AM is the head of the Third Department of Surgery. All authors read and approved the final manuscript.
